# Understanding the role of serological and clinical data on assessing the dynamic of malaria transmission: a case study of Bagamoyo district, Tanzania

**DOI:** 10.11604/pamj.2022.43.60.35779

**Published:** 2022-10-07

**Authors:** Tunu Guntram Mwamlima, Solomon Mickson Mwakasungula, Catherine Gerald Mkindi, Mgeni Mohamed Tambwe, Sarah Senyoni Mswata, Stephen Gabriel Mbwambo, Michael Fred Mboya, Simon John Draper, Sarah Elizabeth Silk, Maxmillian Gideon Mpina, John-Mary Vianney, Ally Ibrahim Olotu

**Affiliations:** 1 Ifakara Health Institute, Bagamoyo, Tanzania,; 2 Department of Life Science and Bio-Engineering, The Nelson Mandela African Institution of Science and Technology, Arusha, Tanzania,; 3 Amref Health Africa-Tanzania, Dar es Salaam, Tanzania,; 4 Department of Biochemistry, University of Oxford, Oxford OX1 3QU, United Kingdom

**Keywords:** Total immunoglobulins (IgG), *Plasmodium falciparum*, anti-schizont antibodies, infants, clinical malaria, malaria transmission

## Abstract

**Introduction:**

naturally acquired blood-stage malaria antibodies and malaria clinical data have been reported to be useful in monitoring malaria change over time and as a marker of malaria exposure. This study assessed the total immunoglobulin G (IgG) levels to Plasmodium falciparum schizont among infants (5-17 months), estimated malaria incidence using routine health facility-based surveillance data and predicted trend relation between anti-schizont antibodies and malaria incidence in Bagamoyo.

**Methods:**

252 serum samples were used for assessment of total IgG by enzyme-linked immunosorbent assay and results were expressed in arbitrary units (AU). 147/252 samples were collected in 2021 during a blood-stage malaria vaccine trial [ClinicalTrials.gov NCT04318002], and 105/252 were archived samples of malaria vaccine trial conducted in 2012 [ClinicalTrials.gov NCT00866619]. Malaria incidence was calculated from outpatient clinic data of malaria rapid test or blood smear positive results retrieved from District-Health-Information-Software-2 (DHIS2) between 2013 and 2020. Cross-sectional data from both studies were analysed using STATA version 14.

**Results:**

this study demonstrated a decline in total anti-schizont IgG levels from 490.21AU in 2012 to 97.07AU in 2021 which was related to a fall in incidence from 58.25 cases/1000 person-year in 2013 to 14.28 cases/1000 person-year in 2020. We also observed a significant difference in incidence when comparing high and low malaria transmission areas and by gender. However, we did not observe differences when comparing total anti-schizont antibodies by gender and study year.

**Conclusion:**

total anti-schizont antibody levels appear to be an important serological marker of exposure for assessing the dynamic of malaria transmission in infants living in malaria-endemic regions.

## Introduction

Malaria is one of the vector-borne diseases of public health significance in sub-Saharan Africa, affecting largely under 5 years [1]. Deployment of the malaria control strategies such as insecticide-treated bed nets (ITNs) [2], indoor residual spraying (IRS) [2], artemisinin-based combination therapies (ACTs) [3], and intermittent presumptive treatment (IPT) [4] have all contributed to the 18% and 28% decline in global malaria cases and deaths respectively between 2010 and 2018 [5]. Nevertheless, the Word Malaria Report of 2021 indicated a slight increase in cases and deaths due to the covid-19 pandemic [6]. Although, the control measures played role in reducing malaria burden, those measures becomes vulnerable to parasite resistance [7,3], and vector resistant [7,8]. Therefore, new control interventions such as vaccines are crucial to complement the current strategies. Generally, vaccines are considered one of the most cost-effective public health intervention for diseases, and have contributed to the elimination of smallpox [9] and reduced childhood mortality [10]. The efficacy and immunogenicity of malaria vaccines candidates could be confounded by exposure [10,11] which modify immune responses [10,11]. Therefore, accounting for malaria exposure to an individual especially in randomized clinical trials that assess time to malaria infection [12] is crucial for improving vaccine efficacy [13].

Approaches for estimating malaria exposure to an individual includes entomological inoculation rates [14], prevalence of malaria within a defined radius of the index participant [15]. These approaches are labour intensive compared to the use of antibodies [16] which is less intensive, but could be influence by age [17] and gender [18]. Assessing antibody levels from historical samples alone to characterize the contemporaneous individual malaria exposure could provide imprecise estimates because malaria exposure correlates with antibody responses [19]. The level of accuracy will depend on how distant in time the historical samples came from, and the changes in malaria transmission during that period. This study, aimed to explore the use of serological data from vaccine trials [20] and archived clinical data from DHIS2 [21] in predicting a trend relation of anti-schizont antibody levels and malaria incidence in under five (infants inclusive) over 8 years period in Bagamoyo district.

## Methods

**Study area:** serum samples and routine health care data were collected within the Bagamoyo district. According to the 2012 Tanzania National Census, the population of the Bagamoyo district was 311,740 [22].The humid tropical climate district has temperatures of 28°C-30°C, humidity up to 98%, and an average rainfall of 1200-2100 mm per year [23]. Rainy seasons occur between March-May and October-December creating temporary and permanent mosquito breeding water ponds that contribute to high malaria infectivity rates during these periods [22].

**Health facility data:** in 2014, Tanzania´s Health Management Information Systems (HMIS) under the ministry of health adopted the use of District Health Information Systems 2 (DHIS2) which is an integrated open-source and web-based platform that works to capture routinely collected data from health facilities [24]. Ten health facilities were selected from seven wards of low (Dunda, Magomeni, Zinga and Kisutu) and high (Fukayosi, Makurunge, and Yombo) malaria transmission areas. The characterization into low and high malaria transmission areas was based on both local/traditional categorization and previous epidemiological studies [25]. Simple random sampling was used to select health facilities for clinical data collection from the wards. Using the DHIS2 and register books, malaria data were retrieved for each selected health facility. The data retrieved included monthly malaria cases by age group (<5, >5-59 and 60+ years) and gender. Population size by age and gender for each ward over 8 years of follow-up was extracted from wards´ executive offices. A malaria case was defined as any patient with confirmed malaria, either by malaria rapid diagnostic test (mRDT) or by blood slide (BS). All health facilities were assumed to be operational throughout the period the data were collected. The DHIS2 data were retrieved, verified at the health facilities and any missed or inconsistence data in the DHIS2 were updated with the actual records from the source.

**Study design:** a cross-sectional study that involved collection of achieved and fresh serum samples from Bagamoyo Clinical Trial Facility of Ifakara Health Institute (BCTF-IHI) and a retrospective data collection from DHIS2.

**Sample collection:** convenient sampling was used to obtain serum samples for serological and simple random sampling for selecting health facility from which clinical data were retrieved. Serum samples for assessment of total IgGs to schizont were from two malaria vaccine trials that were conducted at two different time point from both low and high malaria transmission areas of Bagamoyo district council. 147 freshly collected infants´ serum samples were obtained during the screening process of a candidate blood-stage malaria vaccine trial (RH5.1/MM) conducted in 2021 [ClinicalTrials.gov NCT04318002] [26] and 105 achieved infants´ serum samples from archived malaria vaccine trial (RTS, S/AS01) conducted in 2012 [ClinicalTrials.gov NCT00866619] were obtained from the BCTF-IHI´s biobank. For assessment of total anti-schizont antibodies, malaria negative by BS or mRDT was an inclusion criterial. All samples were pseudo-anonymized to prevent attribution to participant-specific information. Only gender, age and location were extracted from the individual information.

Assessment of total IgG to schizont: Enzyme-Linked Immunosorbent Assay (ELISA) method described by Miura *et al*. [27] was used to determine serum antibody levels. Briefly, schizont extract from *P. falciparum* (3D7 clone produced by Ababacar Diouf, NIH) was used for plate coating as described elsewhere [28]. The stock concentration of 5x10^8^schizont extract /mL was diluted 1 in 1000 to obtain a working concentration of 5x10^2^ schizont/µl using Dulbecco´s phosphate-buffered saline (DPBS) and 50µl of the mixture used for plate coating. ELISA plates were incubated overnight at 4°C to allow antigen binding, blocked by adding 100µl of 5% milk in DPBS and incubated for 1 hour before adding 50µL/well of serum samples, standards, and controls (diluted in 1% milk) in triplicate, and followed by a 2 hours incubation. Thereafter, 50µL of secondary antibody (goat anti-human IgG (?-chain)) was added in each well, incubated for 1 hour and developed with 100µL/well of 1mg/ml developer solution (5x diethanolamine buffer in distilled water and 1x 20mg 4-nitrophenyl phosphate tablet). The plates were developed until assigned development endpoint reached. Extensive washing of ELISA plate was done after each incubation phase. To acquire Optical Density (OD), the plates were read on the Bio-Teck microplate ELx 808TM absorbance reader (Agilent Technologies, UK). ODs acquisitions were done using Gen5 software (Agilent Technologies, UK) at Ab_405nm_wavelength.

**Assay quality control:** assay control was performed as described by Miura *et al*. [27]. Positive control reference at 1 in 16000 dilutions with OD readings at Abs_405nm_threshold of average 0.8 minimum and 1.2 maximum were used. A 2-fold serially diluted standard curve was included in each run. Curve linear portion was Abs_405nm_threshold of average ~0.4 OD for lower limit and ~2.5 OD for upper limit, R^2^ value was >0.994, coefficient of variation (CV) <20%. Blank wells (1% milk) provided Abs_405nm_threshold of average < 0.3 OD. Statistical analysis: data were entered in excel 2013 and imported to STATA version 14 (Stata Corp LP, Texas, USA) for analysis. Descriptive analysis presented median and standard deviation. Kruskal Wallis and Mann-Whitney U test compared median AU between the groups. Age was conveniently categorized into 0-5 months, 6-11 months, and 12-19 months groups, box plots were used for AU and line graphs for malaria incidence trend visualization [29].

Malaria incidences was calculated as follows [30]:


$$Incidence=\frac{confirmedmalariacasesinayear}{mid-yearpopulationxpersonalyear}x1000$$

New cases were defined as the sum of all monthly reported cases in each year. Mid-year population size was defined as the annual mean population size in the areas where malaria cases were obtained. Total population in the health facilities catchment areas were considered at risk of clinical malaria and included in the analysis. Personal years were defined as the time each individual data collected in the study year [31].

**Ethical considerations:** the study received approval from the Ifakara Health Institute-Institutional Review Board (IHI/IRB/No: 49-2020 and 15-2020), National Institute for Medical Research ethical regulatory committee (NIMR/HQ/R.8a/Vol. IX/3537), Oxford Tropical Research Ethics Committee (OxTREC Reference: 9-20), Tanzania Medicines and Medical Devices Authority (Ref. No. TMDA0020/CTR/0005/2) and Local Government Authority (Kumb. Na. JC. 156/254/01).

## Results

### Sampled population for anti-schizont antibody evaluation and for malaria incidence trend estimation

Samples were collected from infant studies conducted in 2012 and 2021. Table 1, shows the demographic information of the sampled population used for analyzing antibodies to *P. falciparum* schizont in infants and Table 2 shows distributions of malaria cases and incidence amongst age, gender and transmission variables.

**Table 1:** the demographic characteristics of the sampled population used for analyzing antibodies to *P. falciparum* schizont in infants

Year	Study	No. of infants (%)	No. of Male (%)	No. of Female (%)	No. of infants from low transmission setting (%)	No. of infants from high transmission setting (%)
	ClinicalTrial.gov NCT00866619	105 (41.7)	48 (45.7)	57 (54.3)	90 (85.7)	15 (14.3)
**2021**	ClinicalTrial.gov NCT04318002	147 (58.3)	78 (53.1)	69 (46.9)	140 (95.2)	7 (4.8)
**Total**		**252**	**126**	**126**	**230**	**22**

**Table 2:** the demographic characteristics, malaria cases and malaria incidence of the study population over the 8 years of follow-up

Variables			Year									
			2013	2014	2015	2016	2017	2018	2019	2020	Total	%Total
**Age**	**<5**	Population size	15,374	16,051	17,110	18,616	20,664	23,391	26,993	31,744	169,943	16
		Cases	1,699	1,636	3,169	2,230	1,023	1,381	1,277	1,935	14,350	28.10
		Incidence	110.51	101.93	185.21	119.79	49.51	59.04	47.31	60.96	734.26	40.45
	**>5-59**	Population size	76,872	80,254	85,551	93,079	103,318	116,956	134,967	158,721	849,717	80
		Cases	3,945	2,842	5,573	4,129	3,182	4,209	3,694	5,305	32,879	64.38
		Incidence	51.32	35.41	65.14	44.36	30.80	36.00	27.37	33.42	323.82	17.84
	**60+**	Population size	3,844	4,013	4,278	4,654	5,166	5,848	6,748	7,936	42,486	4
		Cases	0	71	1182	1101	275	330	278	601	3,838	7.52
		Incidence	0	17.69	276.33	236.57	53.23	56.43	41.20	75.73	757.18	41.71
**Gender**	**Female**	Population size	60,536	63,200	67,371	73,300	81,363	92,103	106,287	124,993	669,152	63
		Cases	2,671	2,109	4,843	3,630	2,193	2,883	2,614	4,442	25,385	48.96
		Incidence	44.12	33.37	71.89	49.52	26.95	31.30	24.59	31.43	313.18	35.86
	**Male**	Population size	35,553	37,117	39,567	43,049	47,785	54,092	62,422	73,409	392,994	37
		Cases	3,047	2,475	5,063	3,668	2,212	2,953	2,563	4,482	26,463	51.04
		Incidence	85.70	66.68	127.96	85.21	46.29	54.59	41.06	52.73	560.22	64.14
**Transmission areas**	**Low**	Population size	71,348	74,487	79,403	86,391	95,894	108,552	125,269	147,316	788,660	74.25
		Cases	4,156	3,764	6,567	4,341	2,966	2,042	1,722	2,104	27,662	54.17
		Incidence	58.25	50.53	82.70	50.25	30.93	18.81	13.75	14.28	319.50	32.67
	**High**	Population size	24,742	25,830	27,535	29,958	33,253	37,643	43,440	51,085	273,487	25.75
		Cases	1,488	785	3,357	3,119	1,514	3,878	3,527	5,737	23,405	45.83
		Incidence	60.14	30.39	121.92	104.11	45.53	103.02	81.19	112.30	658.61	67.33

### Anti-schizont antibody distribution amongst age, gender and malaria transmission intensity

**Figure 1:**
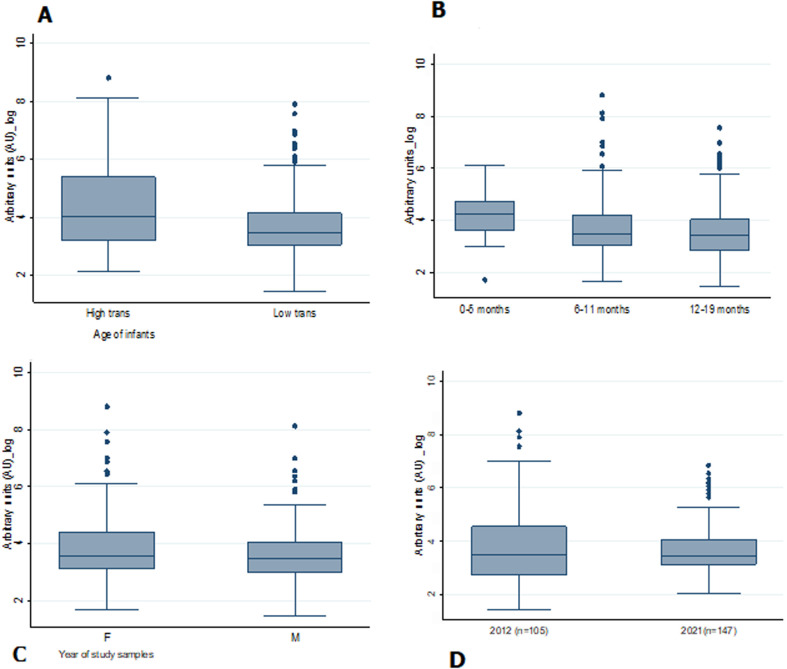
total immunoglobulins G against *P. falciparum* schizont among infants of Bagamoyo in 2012 versus 2021; (1A) by study year, (1B) by gender, (1C) by age group and (1D) by transmission areas; total IgG against *P. falciparum* schizont (Pf3D7) as measured by standardized enzyme-linked immunosorbent assay

**Figure 2:**
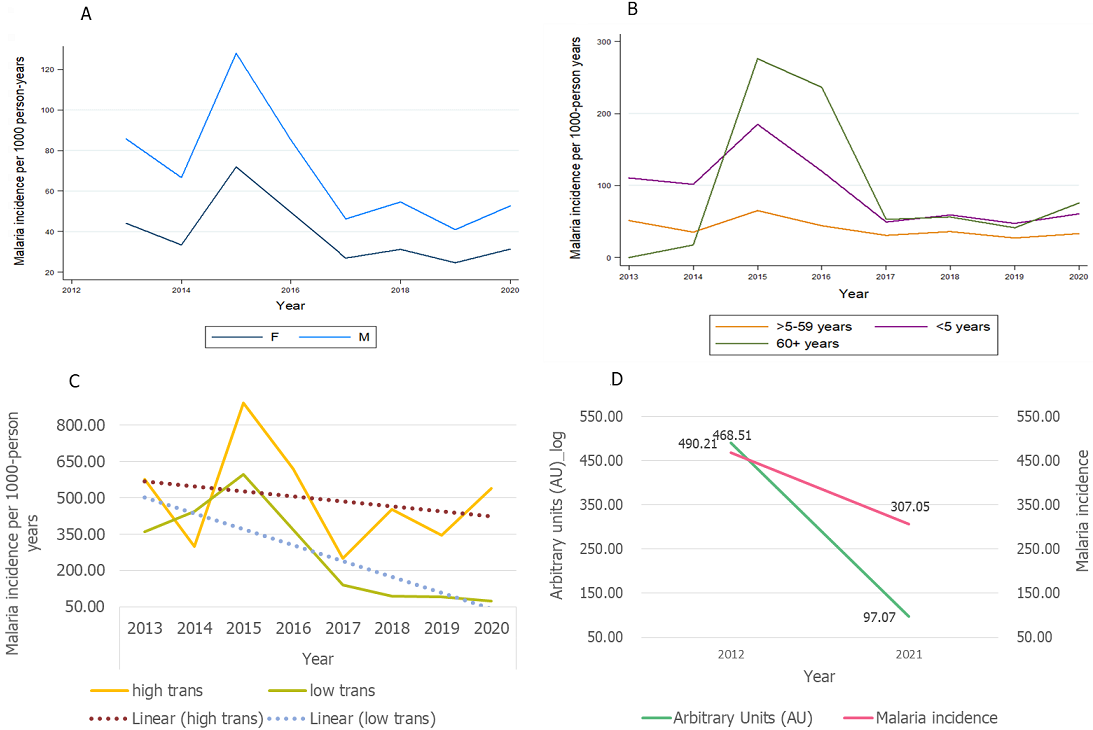
trend of malaria incidence in Bagamoyo for the year 2013-2020, (A) males versus females, (B) by age groups, and (C) by transmission areas, the vertical (y) axis representing malaria incidence and the horizontal (x) axis representing years of follow-up, panel (D) showing the trend of malaria anti-schizont antibodies relative to malaria incidence in under 5 years; M=male, F=female, High trans=high transmission area and Low trans=low transmission area

We showed evidence of a significant difference in levels of anti-schizont antibody when comparing levels by malaria transmission intensity (Figure 1A, p=0.03) and by age (Figure 1B, p=0.02). In addition, a trend positive relation between anti-schizont antibody levels and malaria incidence was observed (Figure 2A). However, there were no significant difference in median (SD) anti-schizont antibody levels during the study period when compared anti-schizont antibody levels by years [33.1AU (829.4) in 2012 and 32 AU (133.1) in 2021] (Figure 1C, p=0.78), and by gender (Figure 1D, p=0.2).

### Eight-year trend analysis of malaria incidence

Overall, we demonstrated a significant decline in malaria incidence in both females and males from year 2015 (78.78 malaria incidence per 1000-person years) to 2020 (42.08 malaria incidence per 1000-person years) (Figure 2B). When categorizing data by age groups, we demonstrated a similar trend of decline in malaria incidence in patients under 5 years and those above 60 years old between 2015 and 2020. However, malaria incidence in patients aged 5-59 years did not change much between the follow-up period (Figure 2C). The current study also showed that, despite starting slightly high in 2013, there was a significant decline in malaria incidence in those under 5 years old from (598.30 malaria incidence per 1000-person years in 2015 to 75.56 malaria incidence per 1000-person years in 2020) in the low malaria transmission area. In high transmission areas, the incidence dropped from 891.30 (in 2015) to 249.89 malaria incidence per 1000-person years (in 2017), followed by a rebound of incidence up to 538.55 malaria incidence per 1000-person years in 2020 (Figure 2D). When comparing anti-schizont antibodies in under 5 patients, we showed a decrease in antibodies against *P. falciparum* anti-schizont was related to a decreased incidence in the respective group (Figure 2A).

## Discussion

We have demonstrated that the level of antibodies to *P. falciparum* schizont in under 5 years depending on the intensity of malaria transmission in Bagamoyo district, Tanzania. In general, we showed that waning in antibodies to *P. falciparum* schizont extract was reflected by a decrease in malaria incidence in under 5 years population, similar to the findings demonstrated in study conducted in Kenya [32]. This study also reported more incidence of malaria in males as compared to females despite, through the follow-up period (year 2013-2020) we observed a decrease in malaria incidence in both groups. The difference in incidence between males and females could be attributed to socioeconomic activities that led to more man exposed to mosquitos when spending time outside their home at night compare to females [33]. In addition, the acceptability of the use of malaria interventions such as treated bed nets has shown to be low in the male population compared to females [34].

Both antibodies and clinical malaria data have widely been used in studies to determine the malaria burden and monitor the impact of interventions over some time [35]. Using serological markers, a robust information on exposure over time and availability of long-lasting antibodies to *Plasmodium spp* can be obtained [35]. Serological data can further provide more insights into malaria epidemiology and help identify hotspots that need targeted interventions [36]. Moreover, the retrospective serological data and clinical data are important and can complement each other in explaining malaria status in malaria-endemic areas [37].

The current study neither observed a general difference in the median anti-schizont antibodies between the infants nor differences when categorized data by gender. This concurs with other studies that have shown no association between gender and malaria antibodies [38]. Previous study have also suggested that antibody distribution in infants may vary according to the level of malaria exposure [39], and/or vary due to level of antibodies they acquired from their mothers while in-utero [40], which is reported to last up to 6 months [41]. A change in anti-schizont antibody levels observed between the years of study samples might be allied to malaria control measures that have been implemented in recent years [42].

Interestingly, the clinical data demonstrated a significant reduction in malaria incidence among those under 5 years over the past 8 years. The outstanding reduction in malaria incidence observed in those under 5 years might also be related to control measures that the National Malaria Control Programs (NMCP) has implemented focusing on this age group [42]. Less malaria incidence seen in the >5-59 years age group, could be due to naturally acquired malaria immunity following several exposures to asymptomatic and symptomatic infections [43]. The >5-59 years age group includes adults that are more active and engage in social activities that put them at risk of contracting malaria parasites [44] resulting in a higher rate of acquisition of naturally acquired immunity [43]. High incidence observed in 60+years age group, could be resulted from the age-related immune compromise [45], thus making them susceptible to infectious diseases including malaria infection [46].

The observed fluctuation in malaria incidence over time in areas of high malaria transmission could be due to unstable implementation of malaria control measures in the region secondary to increased settlements [38]. Additionally, over the last 8 years, there has been a continuous improvement in the access to malaria diagnostic tests and reporting through HMIS providing a more realistic picture of malaria burden in rural Bagamoyo, an area regarded to have high malaria transmission [47]. Furthermore, possible factors that could determine the difference in malaria incidences between areas could be malaria species variation [48], resistance to interventions or efficiency and coverage of interventions [48] as well as social-economic activities [49], host and vector characteristics [49]. A slight trend increase in malaria incidence in 2020 could be a blip or a true increase as a result of the Covid-19 pandemic that might have negatively impacted malaria control programs such as clinical care provision and bed net distribution [6]. However, further studies are required to provide evidence of the impact of Covid-19 on malaria incidence.

The limitations of this study include the availability of a small sample size in a high transmission area for antibody analysis, the use of retrospective hospital data for incidence estimation, and inadequate longitudinal antibody data to conduct a statistical correlation between antibodies levels and malaria incidence. This was due to reliance on cross-sectional samples derived from studies that had different study objectives and the use of secondary clinical malaria data from health care facilities. Further, errors in measurement and reporting from secondary data obtained from health facilities might have reduced data quality [50]. Moreover, meteorological information such as rainfall, temperature, and relative humidity was not included in the analysis. However, serological and clinical results reported by this study are in agreement with findings from other studies, an indication that the above factors did not play a great role in the modification of the current findings [37].

## Conclusion

The present study supports the evidence that the total anti-schizont antibody (IgG) levels depend on malaria transmission intensity in infants of 5-17 months and are positively related to malaria incidence. The use of archived clinical data from DHIS2 and serological data from vaccines or epidemiological studies can potential provide insight into the status of current malaria exposure in areas where proper incidence studies are unavailable.

### What is known about this topic

- Association of antibodies to malaria blood-stage antigens and malaria exposure;
- Use of anti-malaria antibodies to blood-stage antigens in monitoring changes in malaria transmission over time.

### What this study adds

- Further demonstration that total IgG antibody levels to P. falciparum schizont in infants of 5-17 months are related to malaria transmission intensity;
- Assessment of total IgG antibody levels to P. falciparum schizont in infants using both achieved and freshly collected serum samples to understand the change in malaria immunity over time;
- Demonstrate the potential of using antibodies data established from archive samples and hospital data from DHIS to give an insight into malaria exposure that would help better planning of clinical trial studies of malaria vaccine-induced responses in malaria-endemic communities.
